# Questionable research practices among italian research psychologists

**DOI:** 10.1371/journal.pone.0172792

**Published:** 2017-03-15

**Authors:** Franca Agnoli, Jelte M. Wicherts, Coosje L. S. Veldkamp, Paolo Albiero, Roberto Cubelli

**Affiliations:** 1 Department of Developmental Psychology and Socialization, University of Padova, Padova, Italy; 2 Department of Methodology and Statistics, Tilburg University, Tilburg, Netherlands; 3 Department of Psychology and Cognitive Science, University of Trento, Trento, Italy; Universitat Wien, AUSTRIA

## Abstract

A survey in the United States revealed that an alarmingly large percentage of university psychologists admitted having used questionable research practices that can contaminate the research literature with false positive and biased findings. We conducted a replication of this study among Italian research psychologists to investigate whether these findings generalize to other countries. All the original materials were translated into Italian, and members of the Italian Association of Psychology were invited to participate via an online survey. The percentages of Italian psychologists who admitted to having used ten questionable research practices were similar to the results obtained in the United States although there were small but significant differences in self-admission rates for some QRPs. Nearly all researchers (88%) admitted using at least one of the practices, and researchers generally considered a practice possibly defensible if they admitted using it, but Italian researchers were much less likely than US researchers to consider a practice defensible. Participants’ estimates of the percentage of researchers who have used these practices were greater than the self-admission rates, and participants estimated that researchers would be unlikely to admit it. In written responses, participants argued that some of these practices are not questionable and they have used some practices because reviewers and journals demand it. The similarity of results obtained in the United States, this study, and a related study conducted in Germany suggest that adoption of these practices is an international phenomenon and is likely due to systemic features of the international research and publication processes.

## Introduction

Questionable research practices (QRPs) are methodological and statistical practices that bias the scientific literature and undermine the credibility and reproducibility of research findings [1]. Ioannidis [2] famously argued that over 50% of published results are false, and one of the reasons is biases, which he defined as “a combination of various design, data, analysis, and presentation factors that tend to produce research findings when they should not be produced.” Since the start of the current crisis of confidence in psychology [3–5], many scholars have been uncovering direct and indirect evidence of use of QRPs among psychologists. For instance, Franco, Malhotra, and Simonovits [6] compared registered psychological studies with their published results and found that most published studies did not include all the actual experimental conditions and outcome measures. Furthermore, statistically significant results were more likely to be published. A large survey of psychology researchers [7] found that outcome measures and reasons for terminating data collection were often not reported. Also, psychologists report findings that are consistent with their hypotheses more often than in other fields of science [8], and much more often than would be expected based on the average statistical power in psychological studies [9–14]. Evidence has also been found for selective or biased reporting of methods and results [15] that disguises practices such as ad hoc formulation of hypotheses [16], reporting exploratory analyses as confirmatory [17], and other methods that increase chances of publication [9, 18, 19]. In addition, there is evidence of widespread misreporting of statistical results [20, 21].

These studies are evidence that QRPs have biased published psychological research, but it remains unclear how widespread the use of QRPs is among psychologists. Prevalence estimates of engagement in QRPs have primarily been based on studies among scientists from many different fields [22–24]. Recently, two studies have been published of QRP prevalence in psychology. John, Loewenstein, and Prelec [25] surveyed QRP prevalence among psychologists at universities in the United States (US) and found alarmingly high estimates of psychologists who have used QRPs at least once. More than 2,000 psychologists responded to a questionnaire asking whether they had ever employed ten QRPs, their estimations of the percentage of psychologists who have employed QRPs at least once, and how likely they thought it was that other psychologists would admit using QRPs. A ‘Bayesian Truth Serum’ was used: about half the respondents were told that a donation to a charitable organization would be based on the truthfulness of their responses and the other half were a control group for this manipulation. The results were similar for these two groups; on average, psychologists in the experimental group admitted to having used 36.6% of these ten QRPs and psychologists in the control group admitted to having used 33.0% of the ten QRPs. Considering only the self-admission rate in the control condition, six QRPs were used by more than a quarter of the respondents, and of these six, three QRPs were used by about half or more of the respondents (45.8% to 63.4%). Only two QRPs were used by less than five percent of the respondents according to their self-admissions, one of which was to falsify data (which is misconduct rather than merely a questionable practice).

Fiedler and Schwarz [26] conducted a similar survey of members of the German Psychology Association regarding their use of the same ten QRPs, but they substantially modified the wording of the survey and redefined prevalence. In previous research [22–24] and in John et al. [25], prevalence of a QRP was defined as the percentage of people who had engaged in that practice at least once. Fiedler and Schwarz [26] observed that this definition of prevalence does not correspond to the prevalence of a practice in research or in the research literature, because some researchers may have used a QRP only once whereas others may have used it many times. In their research, they [26] estimated prevalence of QRPs in research as the product of two terms: (1) the percentage of people who had engaged in that practice at least once and (2) the rate at which researchers repeated this behavior across all the studies they had conducted. Because John et al. [25] estimated the prevalence of researchers who ever used a QRP and Fiedler and Schwarz [26] estimated prevalence of QRP use in research, their prevalence estimates cannot be directly compared.

Although some researchers have argued that publication pressures are higher among US scientists as opposed to scientists in other countries [27], there is some debate regarding whether biases due to various QRPs are relatively higher in the US [28]. To investigate the extent to which the US prevalence estimates generalize to other countries, we conducted a direct replication of the US study among Italian research psychologists. Because US and Italian researchers all participate in the international research community, we expected that QRP prevalence would be similar in the two countries. There are, however, cultural and organizational differences between academia in these countries and differences in the participant sampling of our studies, possibly resulting in some differences in practices, but we had no specific predictions about the size or direction of these differences.

## Materials and methods

The questionnaire used by John et al. [25] was translated into Italian by three native speakers of Italian with expertise in psychology, including two authors of this paper. The three translators met and agreed upon a common translation. A native English speaker with expertise in psychology performed a back translation. The translated questionnaire is included in the supporting information (S1 Appendix). The translated questionnaire was implemented using the survey software Qualtrics and the anonymous responses were collected through a Tilburg University web server. The data were downloaded as a spreadsheet and analyzed in Excel.

An invitation email was sent to the 1,167 members of the mailing list of the Italian Association of Psychology (AIP) in October, 2014. AIP is an association of psychologists involved in research at universities or research centers in Italy. The email message invited them to participate in a survey and provided a link to the questionnaire. The mailing list included 802 dues-paying AIP members for the year 2014. There were 277 respondents (24% response rate) who answered at least part of the questionnaire and 208 respondents (75% of the 277 respondents) completed it.

Before beginning the questionnaire, participants were informed of the purpose of the study, assured that their responses would be anonymous and that locations of the respondent could not be traced, and informed that they could stop their participation at any time. To continue, they registered their informed consent to participate by clicking a button. This procedure follows the ethical standards of the American Psychological Association [29] and was approved by the AIP executive board and by an ethics committee of the University of Padova.

The questionnaire included 10 QRPs presented in an order randomized for each participant. Four questions were asked for each QRP. First, participants were asked to estimate the percentage of Italian research psychologists who had ever employed the QRP (prevalence estimate). Second, they were asked to consider the Italian research psychologists who had employed the QRP and estimate the percentage that would state that they had done so (admission estimate). Third, participants were asked whether they had personally ever adopted this research practice (self-admission), with response options “yes” and “no”. Fourth, they were asked whether they thought that employing the QRP is defensible, with response options “no”, “possibly”, or “yes”. At the end of the questionnaire we included additional questions, described below, concluding with the option to leave comments or suggestions in an open text box.

Because John et al. [25] found little difference between the results from the ‘Bayesian Truth Serum’ condition and the results from the control condition (and because of the extra cost to implement the ‘Bayesian Truth Serum’ condition), we did not use the ‘Bayesian Truth Serum’ in our study. The section of the survey described above thus constitutes a direct replication of the design used in the control group in the study by John et al. [25], but now conducted with Italian research psychologists.

## Results

Data from all 277 respondents were included in the analysis, including those who did not complete the questionnaire, because the QRPs were presented in a random order. The anonymous data are available at https://osf.io/6t7c4/.

### Self-admission rates and defensibility

Table 1 presents the self-admission rates and their confidence intervals for the US academic psychologists in the control group of [25] and for the Italian research psychologists in our sample. The US confidence intervals were computed from data provided by Leslie John, an author of [25]. Self-admission rates across QRPs were similar among the American and Italian research psychologists and were highly correlated, *r* = .94, 95% CI [.76; .99]. The mean self-admission rate across all ten QRPs was 27.3% in Italy and 29.9% in the US. Differences between the US and Italian self-admission rates can be evaluated by comparing their confidence intervals. Of course, interpreting these comparisons requires assuming that both samples are either not affected by selection biases or are affected by selection biases in very similar ways. The US self-admission rates were significantly higher than the Italian rates for QRPs 1 and 3. The Italian self-admission rate was significantly higher only for QRP 8. Nearly all research psychologists (88% in Italy and 91% in the US) who finished the survey reported having employed at least one QRP. Few US and Italian respondents admitted ever falsely claiming that results were unaffected by demographic variables (QRP 9) or falsifying data (QRP 10). The low self-admission rates for QRP 9 (3.0% US and 3.1% Italian) may reflect low frequencies of research examining demographic variables and consequently few opportunities to engage in this practice. We are not surprised that few respondents admitted falsifying data, but we find it disturbing that three US and five Italian researchers admitted it.

**Table 1:** Questionable Research Practices (QRPs) and self-admission rates in percentages for US [25] and Italian psychologists.

	US		Italian Association of Psychology	
QRP	Self-admission rate (*N*)	95% CI	Self-admission rate (*N*)	95% CI
1. In a paper, failing to report all of a study’s dependent measures	63.4 (486)	59.1–67.7	47.9 (219)	41.3–54.6
2. Deciding whether to collect more data after looking to see whether the results were significant	55.9 (490)	51.5–60.3	53.2 (222)	46.6–59.7
3. In a paper, failing to report all of a study’s conditions	27.7 (484)	23.7–31.7	16.4 (219)	11.5–21.4
4. Stopping collecting data earlier than planned because one found the result that one had been looking for	15.6 (499)	12.4–18.8	10.4 (221)	6.4–14.4
5. In a paper, “rounding off” a *p* value (e.g., reporting that a *p* value of .054 is less than .05)	22.0 (499)	18.4–25.7	22.2 (221)	16.7–27.7
6. In a paper, selectively reporting studies that “worked”	45.8 (485)	41.3–50.2	40.1 (217)	33.6–46.6
7. Deciding whether to exclude data after looking at the impact of doing so on the results	38.2 (484)	33.9–42.6	39.7 (219)	33.3–46.2
8. In a paper, reporting an unexpected finding as having been predicted from the start	27.0 (489)	23.1–30.9	37.4 (219)	31.0–43.9
9. In a paper, claiming that results are unaffected by demographic variables (e.g., gender) when one is actually unsure (or knows that they do)	3.0 (499)	1.5–4.5	3.1 (223)	0.9–5.4
10. Falsifying data	0.6 (495)	0.0–1.3	2.3 (220)	0.3–4.2

Note: Confidence intervals for US psychologists were computed from data provided by Leslie John.

John et al. [25] reported that QRP self-admission rates for US academicians approximated a Guttman scale [30]. Respondents who admitted to using a relatively rare practice (e.g., stopping data collection after achieving the desired result) had usually used more common practices. Consistency with a Guttman scale is measured by the coefficient of reproducibility, which is the average proportion of a participant’s responses that are predictable by simply knowing the number of affirmative responses (see [31]), and this coefficient was .80 for the self-admission rates of US academicians [25]. The self-admission rates for the 208 Italian research psychologists who responded to all 10 QRPs also approximated a Guttman scale; the coefficient of reproducibility was .87. As John et al. [25] observed, this consistency with a Guttman scale suggests that “there is a rough consensus among researchers about the relative unethicality of the behaviors, but large variation in where researchers draw the line when it comes to their own behavior” (p. 527).

Although self-admission rates were similar in both countries, judgments about QRP defensibility (shown in Table 2) differed considerably. Possible defensibility responses were “no”, “possibly”, and “yes”. In [25] these responses were assigned values of 0, 1, and 2, respectively, and then averaged to obtain a mean defensibility rating for each QRP. In Tables 1 and 2, QRPs are listed in the order of decreasing defensibility ratings for the US psychologists. The defensibility response options constitute an ordinal scale, however, and consequently a mean rating is an inappropriate metric. Instead, Table 2 presents the distributions of responses for US and Italian psychologists and chi-square tests comparing the US and Italian distributions.

**Table 2:** Distributions of defensibility response percentages for US [25] and Italian psychologists.

	Self-admitted US respondents				Self-admitted Italian respondents					All other Italian respondents				
QRP	*N*	No	Pos	Yes	*N*	No	Pos	Yes	χ^2^	*N*	No	Pos	Yes	χ^2^
1	274	1	10	89	105	6	60	34	120.4	114	59	38	4	279.7
2	261	1	21	78	118	3	42	55	21.0	104	28	59	13	146.8
3	127	0	17	83	36	14	72	14	67.7	183	68	31	1	238.4
4	70	3	16	81	23	9	70	22	27.9	198	51	42	7	152.6
5	104	4	22	74	49	16	47	37	20.9	172	78	19	3	181.7
6	202	3	26	71	87	14	66	21	62.8	130	62	37	2	191.9
7	169	5	25	70	87	9	82	9	86.1	132	70	29	1	182.6
8	119	8	35	57	82	9	73	18	31.5	137	70	27	3	128.7
9	12	0	50	50	7	43	29	29		216	92	7	0	
10	3	100	0	0	5	60	20	20		215	99	1	0	

Note: The US defensibility responses are for the control condition only and were provided by Leslie John. Pos indicates the response “Possibly”.

John et al. [25] asked only those researchers who admitted using a QRP to assess whether their actions were defensible, whereas we asked *all* respondents whether employing the QRP was defensible. The middle section of Table 2 (the columns labeled “Self-admitted Italian respondents”) presents the defensibility response percentages for only those Italian psychologists who admitted using the relevant QRP. The section labeled “All other Italian respondents” presents the defensibility response percentages for Italian psychologists who did not admit using the QRP. The US defensibility response distributions are consistently and significantly different from the responses of those Italians who admitted using the QRP and different from the responses of Italians who did not admit using the QRP, resulting in χ^2^ statistics with 2 degrees of freedom much greater than the criterion value of 5.99 with α = .05. No χ^2^ statistics were computed for QRPs 9 and 10 because few US and Italian psychologists admitted having ever used these two practices.

For QRPs 1 through 8, relatively few psychologists who admitted using a QRP responded “No”, that it was not defensible (less than 10% of US psychologists and less than 20% of Italian psychologists). For all eight of these QRPs, US psychologists who admitted using a QRP most frequently responded “yes”, that this practice was defensible. Instead, Italian psychologists who admitted using the QRP most frequently responded “possibly” for all but one of these eight QRPs. Most Italian researchers (55%) who admitted having decided whether to collect more data after checking whether the results were significant (QRP 2) responded that it is a defensible practice.

The defensibility response for Italian psychologists who did not admit using a QRP (the right-most section of Table 2) was most frequently “No” for seven of QRPs 1 through 8 and “Possibly” only for QRP 2. Again, the striking exception is QRP 2, with only 28% responding “No”, that it is not defensible to collect more data after a failure to obtain a significant result. More than half the Italian psychologists admitted using this QRP, and only 31 of 222 respondents responded that it is not a defensible practice, suggesting that the statistical consequences of this practice [32] are not well known in this sample. The responses of Italian psychologists who did not admit using a QRP indicate that they view these eight QRPs as much less defensible than the US and Italian psychologists who admitted using them.

### Prevalence estimates

The first three questions asked by John [25] and this replication provide three different ways to estimate QRP prevalence, which John et al. [25] defined as the percentage of researchers who have employed the QRP at least once. First, respondents estimated prevalence based on their knowledge and experience within the Italian research community (prevalence estimate). Second, respondents estimated, among Italian research psychologists who had ever employed a QRP, the percentage that would admit to having done so (admission estimate). This is not a prevalence estimate, but John et al. [25] used it to calculate one as explained below. Third, respondents reported whether they had ever employed the QRP in their own research (self-admission). Self-admission rates are likely to underestimate actual prevalence because it is likely that some researchers who have employed a QRP would not admit it. Dividing the self-admission rate by the admission estimate (as in [25]) yields a third prevalence estimate that corrects for the percentage of respondents who employed but did not admit using a QRP.

As Fiedler and Schwarz [26] observed, two of these estimates depend on participants knowing what others in the research community do. Researchers have immediate knowledge of their own behaviors, but they cannot know in detail how others conduct their own research or what they would say about it in a survey. We therefore believe that, although the prevalence estimate and admission estimate offer interesting insights into the opinions of researchers about the behaviors of other researchers, their validity as estimates of behavior is questionable. Nonetheless, we calculated these estimates to permit comparisons with the prevalence estimates obtained by John et al. [25].

**Fig 1:**
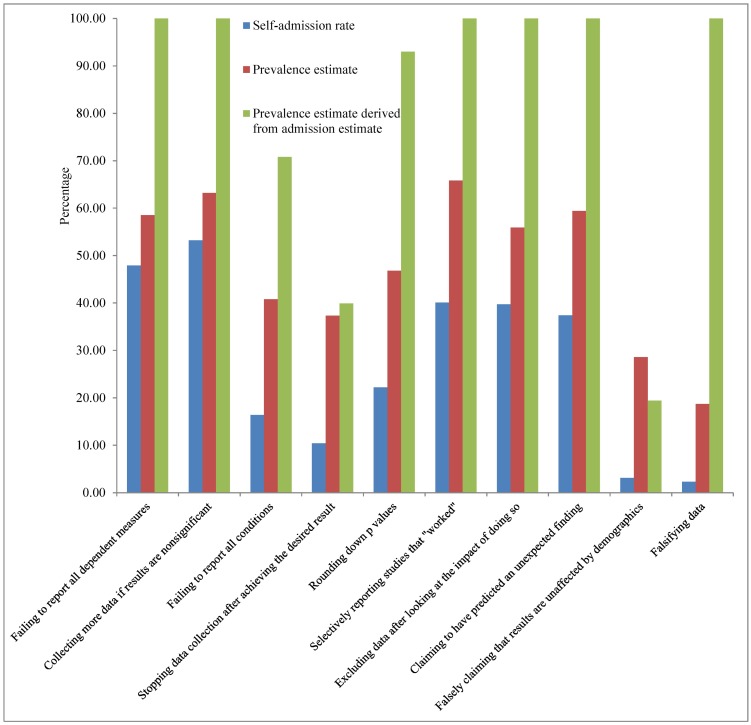
Self-admission rate, prevalence estimate, and the derived prevalence estimate computed by dividing self-admission rate by the admission estimate for each QRP.

Fig 1 presents the QRP self-admission rates (mean = 27.3%), the respondents’ prevalence estimates (mean = 47.5%), and the derived prevalence estimates obtained by dividing self-admission rates by the respondents’ admission estimates (mean = 82.3%). The overall pattern of these three estimates of prevalence are similar in many respects to the estimates reported in Fig 1 of John et al. [25] for US academic psychologists. For both Italian and US respondents, derived prevalence estimates were generally largest (often greater than 90%), self-admission rates were generally smallest, and both the self-admission rates and prevalence estimates increase and decrease from one QRP to another in roughly the same way. Although the overall patterns are similar, there are significant differences in magnitudes. Italian participants’ prevalence estimates (mean = 47.5%) are substantially and consistently greater than the US prevalence estimates (mean = 39.1%), and consistently greater than Italian self-admission rates, suggesting that Italian researchers suspect that these QRPs have been used by more members of the Italian psychological research community than would be estimated based on self-admissions. For example, they estimated that 18.7% of Italian psychology researchers have falsified their data at least once, but only 2.3% admitted having done so.

As in John et al. [25], the third prevalence estimate is obtained by correcting the self-admission rates for the estimated likelihood that a psychologist who had used a QRP would admit it. This derived prevalence estimate is computed by dividing self-admission rates by participants’ mean admission estimates. Averaging over QRPs, the mean admission estimate was only 27%, almost identical to the mean self-admission rate, implying that participants expected that researchers who use these practices would be unlikely to admit it. Dividing self-admission rates by these low admission estimates yielded very large derived prevalence estimates for most QRPs, with estimates greater than 90% for seven of the ten QRPs. These ratios can and did exceed 100% and were capped at that level. Similarly, John et al. [25] obtained four derived estimates of 100% for US psychologists. We consider these derived prevalence estimates unrealistically large and conclude, instead, that admission estimates are not valid measures of the probability of admitting the behavior. Consequently, QRP prevalence estimates derived from admission estimates are not valid (see also [26]). We also note that the variance of derived prevalence estimates is the sum of the self-admission-rate variance and the admission-estimate variance, and consequently the derived estimates are least reliable even if they were valid.

### Doubts about research integrity

**Fig 2:**
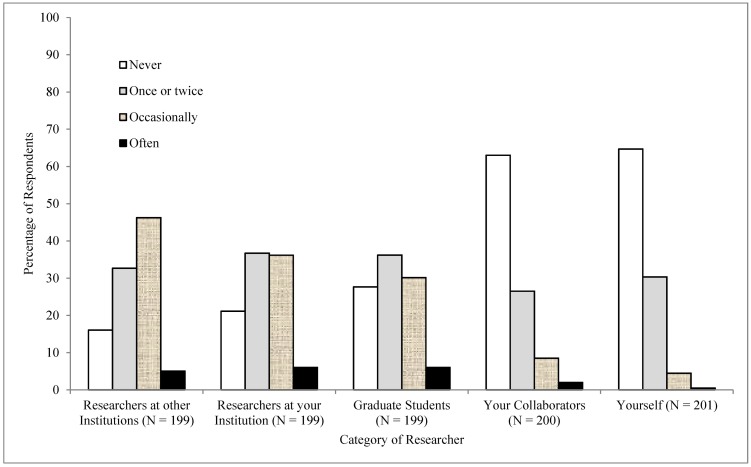
Distribution of responses regarding doubts about the integrity of other researchers and themselves.

After responding to the questions about QRPs, respondents were asked whether they ever had doubts about their own integrity and the integrity of ‘your collaborators’, ‘graduate students’, ‘researchers at your institution’, and ‘researchers at other institutions’. For each category of researcher, respondents indicated whether they had doubted their integrity ‘never’, ‘once or twice’, ‘occasionally,’ or ‘often’. Fig 2 presents the distribution of responses for each category of researcher. Again, the results are similar to the doubts expressed by US researchers reported in Fig 2 of John et al. [25]. A large percentage of respondents in both countries reported having occasional doubts about the integrity of researchers at other institutions, their own institutions, and graduate students. About 35% of Italian researchers and 31% of US researchers reported having had doubts about their own integrity on at least one occasion. Most respondents have great faith in the research integrity of their collaborators and themselves, but about half the respondents (51% Italian and 49% US) occasionally or often have doubts about the integrity of researchers at other institutions.

### Temptations

At the end of the questionnaire we asked some questions that had not been asked by John et al. [25]. Respondents were asked whether they had been tempted to adopt one or more of these QRPs in order to augment their chances of either publishing or career advancement during the past year. Of 202 responses to this question, 53% said they had never been tempted, 37% said they had been tempted once or twice, 7% said they had occasionally been tempted, and 3% said they had often been tempted.

### Demographics of respondents

Respondents were asked to identify their division of the Italian Association of Psychology (AIP) and their career position. The five AIP divisions are self-selected, and correspond to primary areas of research. The number of respondents, their mean rate of self-admission, and their defensibility responses are presented in Table 3. The highest self-admission rates were from researchers in social, experimental and organizational psychology. Developmental, educational and clinical psychologists reported lower rates. Clinical psychologists also were the least likely to say that a QRP was defensible.

**Table 3:** Number of participants and mean self-admission rate and defensibility responses by demographic category.

Demographic category	*N*	Self-admission rate (%)	Defensibility (%)		
			No	Possibly	Yes
AIP Division					
Clinical	33	23.6	63	32	5
Organizational	21	28.6	48	38	14
Developmental & Education	48	22.5	55	34	11
Social	47	30.6	53	34	13
Experimental	54	29.6	60	31	9
Career position					
Post-docs & PhD students	37	25.7	54	36	10
Assistant Professors	63	25.9	54	35	11
Associate Professors	38	26.3	62	30	7
Full Professors	37	28.4	61	27	12
Others	24	30.0	51	42	7

Self-admission rates increased monotonically with academic career position, although the differences between levels are small. Of course, people more advanced in their careers have had more opportunities to engage in a QRP. There were no notable or systematic differences in defensibility responses across career positions.

### Qualitative analysis of respondents’ comments

The questionnaire concluded with an invitation to write a comment, suggestion, or question in a text box. There were 52 respondents who entered text, and their entries ranged in length from 1 to 316 words. We analyzed these texts using the open coding method of grounded theory [33], a qualitative method for identifying content categories. Eight categories of content were identified, and two analysts independently sorted all entries into one or more of these categories. There were a few differences in their sorting, which were reconciled in discussions. The 52 responses yielded 80 instances of the eight categories. Table 4 lists the eight categories and the number of instances of each category in the text.

**Table 4:** Categories and the number of text entries assigned to each category.

Categories	Number of instances
1. QRP is okay	15
2. Example	10
3. Culture	12
4. Compliment	5
5. Ambiguity	13
6. Suggestion	12
7. Limitations	7
8. Estimation difficulty	6

Categories 1 through 3 include comments about QRPs, whether they are appropriate, and why they are used. In 15 instances respondents wrote that one or more of the QRPs were not inappropriate in research. For example, one respondent wrote “I think some of these practices do not constitute by themselves a violation of research integrity.” (Note: this and all other quotes of respondents were translated to English from the original Italian.) In 10 of the 15 responses containing a Category 1 instance, respondents included an example (Category 2) in which a QRP was not inappropriate in their judgment. One respondent wrote, for example, “To search for outliers post hoc is justifiable when dealing with unforeseen results in order to comprehend the reason.” Twelve respondents explained their use of QRPs by reference to demands of the research culture (Category 3), such as the requirements imposed by journals or reviewers. For example, one respondent wrote, “These are practices often required by reviewers of scientific journals (for example, nobody publishes studies that have not obtained any significant results; sometimes reviewers ask that hypotheses be inserted that were not foreseen at the time of submission, other reviewers require not citing variables that did not lead to significant results)”. These voluntary statements suggest that many members of the Italian research community were unaware of the negative consequences of using some of these QRPs or believed that their use was required for publication.

Categories 4 through 8 were about the survey itself or the experience of participating in it. A few text entries were simple compliments (e.g., “interesting”), complaints (e.g., “boring”), or requests for the results (Category 4). In a fourth of the 52 text entries, respondents complained that some of the questions were ambiguous, leaving them uncertain how to respond (Category 5). Indeed, the ambiguity of some questions in the survey was a principal criticism made by Fiedler and Schwarz [26], who obtained lower self-admission rates for questions reworded to reduce ambiguity. Twelve responses included suggestions for revising the questionnaire or its analysis, often suggesting more opportunities for text boxes to explain responses (Category 6). Seven respondents argued that the study results are limited to a certain kind of research (Category 7). One wrote, for example, “The questions refer exclusively to quantitative research methods. Other methods (qualitative, mixed methods, case studies, etc.) are not considered.”

Six respondents wrote about the difficulty of estimating the percentages of other researchers who employ QRPs or who would admit that they do (Category 8). One respondent wrote, “I found it absolutely impossible to estimate the percentage of other researchers who have adopted or declared to have adopted the various practices. I think that any response is arbitrary.” Another respondent wrote, “It is impossible to define the percentage of researchers who adopt these methods.” These complaints strengthen our belief that the QRP prevalence estimate and the prevalence estimate derived from admission estimates may be informative about beliefs but are not valid estimates of the actual prevalence.

## Discussion

US psychologists [25] and Italian research psychologists admitted having used QRPs to a similar extent. However, Italian psychologists less often reported failing to report all a study’s dependent measures and study conditions. Italian psychologists more often reported an unexpected finding as having been predicted. The reasons for these differences between US and Italian self-admission rates are not obvious and could be partly due to sampling biases inherent in surveys with voluntary participants or to the translated statements not being measurement invariant across both samples.

The differences across QRPs in self-admittance rates were also very similar for US and Italian psychologists. Self-admittance rates in both countries approximated a Guttman scale, and the self-admittance rates of US and Italian psychologists were very highly correlated (*r* = .94). These findings indicate that the problem of QRP usage by research psychologists is not limited to the US, but is also a problem in Italy.

John et al. [25] found that US psychologists who admitted having used a QRP most frequently responded that its use was defensible and rarely responded that it was not defensible. Italian research psychologists who admitted using a QRP most frequently responded that its use was possibly defensible. Italian psychologists who did not admit using a QRP most frequently responded that the practice was not defensible and rarely responded that it was defensible. These responses suggest that Italian psychologists, even psychologists who used these practices, consider these practices to be questionable.

Employing the methods used by John et al. [25], we obtained three estimates of the prevalence of Italian psychologists who have ever used each QRP, but none of these three is a precise estimate of prevalence. The self-admission rate (mean = 27.3%) is likely to be an underestimate of the actual rate because some researchers will not admit having used the practice. The magnitude of this underestimation is unknown, but the Bayesian truth serum manipulation employed by [25] increased the self-admission rate by only 3.6%, suggesting that the overall difference between self-admission rates and actual prevalence in the population is not large. The difference between self-admission rates in the Bayesian truth serum manipulation and control condition in [25] was, however, larger for “more questionable” QRPs, suggesting that self-admission rates underestimate the actual prevalence more for these QRPs.

Much larger prevalence estimates were derived from respondents’ estimates of the behaviors of other Italian psychologists. They were asked to estimate the percentage who used each QRP (mean = 47.5%) and estimate the percentage who would admit doing it. A derived prevalence estimate (82.3%) is obtained by dividing this admission estimate into the self-admission rate. As Fiedler and Schwarz [26] noted, the validity of these two metrics (prevalence estimate and derived prevalence estimate) for estimating actual prevalence in the population is open to question. Researchers have some knowledge about the acceptability of QRPs within their organizational milieu but are unlikely to have detailed knowledge about other psychologists’ QRPs or their willingness to admit these behaviors, and comments from our participants confirmed that some lacked the information required for an informed response.

Although these metrics do not provide accurate estimates of actual QRP prevalence in the population, our respondents’ prevalence estimate and admission estimate are informative about beliefs regarding research practices. They indicate a belief within the research community that Italian research psychologists are likely to use these QRPs but very unlikely to admit doing it. Furthermore, these beliefs appear to reflect the relative frequency of QRP use as measured by self-admission rates, which are highly correlated with both prevalence estimates (.94) and admission estimates (.88). Similarly, the US self-admission rates in the control condition of [25] were highly correlated with both prevalence estimates (.92) and admission estimates (.90).

Fiedler and Schwarz [26] replicated the John et al. study [25] among members of the German Psychological Association, but with substantial changes in the QRP descriptions and the procedure. They retained the same ten QRPs but reworded some of them to reduce ambiguity and narrow their scope. They also changed the answer options: they asked their participants whether they had ever engaged in each QRP and, if the answer was yes, in what percentage of all their published findings they had done so. The product of the percentages obtained from these two questions provides an estimate of the prevalence of each QRP in published research. Not surprisingly, the estimated prevalence in published research (mean = 5.2%, see Prevalence 1 in Fig 2 of [26]) is much lower than their self-admission rate (mean = 25.5%), which estimates the prevalence of researchers who have ever engaged in these behaviors.

Fiedler and Schwarz mistakenly concluded that self-admission rates were much lower for German psychologists than for US psychologists. Instead of comparing the German self-admission rates with US self-admission rates, they compared the German rates with the geometric means of the three US prevalence estimates, which were much larger than the US self-admission rates. Not surprisingly, the self-admission rates for the QRPs reworded by Fiedler and Schwarz [26] were lower than the rates reported by John et al. [25], but despite the decrease in rates for the reworded QRPs, the overall self-admission rates are similar for US (29.9%), Italian (27.3%), and German (25.5%) researchers. One possible reason for lower-self admission rates in Italy and Germany is that these surveys occurred later, after publication of John et al. [25] and the onset of discussions about the reproducibility crisis. It should be remembered, however, that the Italian survey was conducted in 2014, just two years after publication of John et al. [25], and the same year that the first paper was published about this topic in a major Italian journal [34].

In all three surveys (in the US, in Germany, and in Italy) participants admitted having used more than a quarter of these ten QRPs on average, and both the US and Italian participants who used the QRPs considered these practices to be defensible or possibly defensible. These consistent results are substantial reasons for concern about the integrity of the research literature. As Fiedler and Schwarz [26] observed, however, an admission of having engaged in a behavior at least once should not be considered an estimate of prevalence of that QRP in their research. Nonetheless, the high defensibility ratings suggest that researchers do not see the harm in the use of many of these practices and may consider them to be acceptable practices.

Adoption of any of these QRPs by a substantial percentage of researchers has serious consequences for the published research literature. Simmons et al. [18] demonstrated that researchers can easily obtain statistically significant but non-existent effects by use of a few of these QRPs, including failing to report all dependent measures, deciding whether to collect more data after seeing whether the results were significant, and failing to report all the conditions of a study. As Table 1 shows, about half the Italian psychology researchers reported having engaged in the first two of these QRPs. The use of such QRPs in studies also severely inflates the estimates of actually existing effects, thereby biasing meta-analyses and obscuring actual moderation of effects [9, 35].

Four of these ten QRPs (QRPs 2, 4, 5, and 7 in Table 1) are very closely related to the use of the null hypothesis significance testing paradigm. The high US and Italian defensibility ratings for these four QRPs (see Table 2) suggest that many researchers are insufficiently aware of the inherent problems in these practices. People have great difficulty with statistical reasoning and the cognitive distortions that it engenders (see [36]). Consider the 5^th^ practice, rounding off a *p-*value. Researchers in the US, Italy, and Germany had remarkably consistent self-admission rates of 22% for the practice of rounding off *p*-values. Of course, the opportunity to obtain an apparently significant result by incorrectly rounding off a *p*-value does not occur in every study, but its practice can be detected by comparing the actual *p*-value for a reported test statistic with the reported *p*-value. Large scale evidence indicates that incorrectly reported results that bear on significance appear in over 12% of psychology articles that use significance testing [20, 21] and similar rates of misreporting appear in a premier Italian psychological journal [37]. Other direct evidence of QRP use among psychologists is based on disclosures by authors themselves [7] and on comparing materials used in studies with the later articles that report them [6].

Other QRPs (QRPs 1, 3, 6, and 8) are associated with decisions about what to include in a research paper. Twenty-three percent of the comments written by Italian research psychologists included explanations for having engaged in one or more of these QRPs because of demands of the publication process. They said that journals, editors, or reviewers had required that they eliminate uninteresting dependent measures or study conditions, that they only report the experiments with significant results, or that the introduction be rewritten to predict an unexpected finding.

Falsifying data is the most egregious of these practices, and cannot properly be considered a questionable practice. There should be no question that it is unethical. Nonetheless, 2.3% of Italian research psychologists reported falsifying data as shown in Table 1. Similarly, 0.6% of US academic psychologists (Table 1 of [25]) and 3.2% of German psychologists [26] reported falsifying data. These results align with the wider literature on the prevalence of falsification of data based on surveys of researchers in many other scientific fields [23].

The high rates of self-admitted QRP use in the US, Germany, and Italy are alarming. The consistency of these rates across all three countries is evidence that QRPs are due to systemic problems in international research and publication processes. O’Boyle, Banks, and Gonzalez-Mulé [15] “posited that the current reward system and absence of checks and balances in the publication process create a strong motive and opportunity for researchers to engage in QRPs as a means to better their chances of publication” (p. 14–15). If, indeed, this is a systemic problem, it cannot be solved by asking or expecting researchers to be more careful or more ethical.

False findings and biases in the literature are generally seen as having three interrelated causes. First, there are increasingly strong pressures in academia to publish research articles [38]. Second, editors and reviewers of journals are widely thought to be more likely to accept papers that report statistically significant results [39]. Third, researchers can make choices when conducting, analyzing, and reporting research that increase the probability that results will be statistically significant. Indeed, Simmons et al. [18] observed that “it is unacceptably easy to publish ‘statistically significant’ evidence consistent with *any* hypothesis” (p. 1359). Researchers who choose to adopt questionable research practices (QRPs) have additional degrees of freedom. These practices increase the likelihood of finding evidence in support of a hypothesis, but the evidence may be spurious or exaggerated. Researchers who adopt these practices are more likely to achieve statistically significant results, which are more likely to be publishable, giving these researchers an advantage in the competition for publications and its rewards. Indeed, perceived pressure to publish is positively related to admission of using unaccepted research practices in economics [40].

As van Dalen and Henkens [41] observed, greater pressures to publish are associated with more frequent publications, and countries worldwide have been adopting metrics and procedures intended to increase pressure to publish. Fanelli [39] observed that the frequency of publishing negative results has been diminishing in most countries, suggesting that a publication bias is growing stronger. European researchers are in direct competition with US academicians because recently adopted European academic evaluation systems reward publishing in international English-language journals. In Italy, for example, the Italian National Scientific Qualification was introduced in 2010 in a reform of the national university system [42]. This qualification system defines bibliometric criteria for advancement that include the number of journal publications, citation counts, and h-index (see [43]). Researchers seeking advancement will certainly be motivated to maximize their scores on these criteria.

Solutions to these systemic problems will require a greater emphasis on the quality of research instead of its quantity (see [44, 45]). As Banks, Rogelberg, Woznyj, Landis, and Rupp [46] argue, action is clearly needed to improve the state of psychological research. Recent papers have suggested steps that would introduce greater transparency in the research process with respect to data [47], collaboration [48], research materials [49], and analyses [50]. The prior specification (pre-registration) of research hypotheses and detailed analyses plans [50–54], heightening the power of studies [9, 53], and reviewers being more open to imperfect results [54] will all help lower QRP use and will eventually increase reproducibility and replicability of research findings in psychology.

## Supporting information

S1 Appendix Questionnaire in Italian. (PDF)
