# Supplementary material for: Questionable research practices among italian research psychologists
Source: PLoS One. 2017 Mar 15;12(3):e0172792. doi: 10.1371/journal.pone.0172792 (PMC5351839; doi:10.1371/journal.pone.0172792)
Supplement: S1 Appendix — (PDF) [file pone.0172792.s001.pdf]

S1 Appendix. Questionnaire in Italian

La presente ricerca fa parte di un più ampio progetto che coinvolge studiosi di altri paesi europei e si propone di indagare prassi di ricerca che sono oggetto di discussione e controversia, ma di cui non si conosce la diffusione tra gli psicologi.

Il questionario online al quale Lei accederà Le chiederà di stimare la percentuale di psicologi ricercatori italiani che hanno adottato le suddette pratiche e di dichiarare se anche Lei le ha adottate, anche solo occasionalmente.

Il questionario è inviato a tutti gli psicologi iscritti all'Associazione Italiana di Psicologia che costituiscono una comunità ampia e rappresentativa della ricerca psicologica italiana.

Il questionario online è stato programmato da colleghi dell'Università di Tilburg (Olanda) in modo da assicurare l'assoluto anonimato dei partecipanti e la non tracciabilità del luogo di origine da cui è partita la risposta. In nessun caso sarà possibile acquisire dati personali o risalire all'identità della persona che ha risposto.

Il questionario è composto da 10 domande e richiede pochi minuti per essere completato. Se accetta di compilare il questionario ha la possibilità in qualsiasi momento (e per qualsiasi motivo) di interrompere la compilazione, ritirandosi in questo modo dalla ricerca con la certezza che i Suoi dati non saranno registrati e utilizzati.

I dati ricavati dalla ricerca saranno trattati esclusivamente a livello di gruppo e saranno unicamente oggetto di comunicazione scientifica.

Per ulteriori informazioni relative alla ricerca potrà rivolgersi al responsabile scientifico, Prof. Franca Agnoli, al seguente indirizzo di posta elettronica: franca.agnoli@unipd.it

Cliccando il pulsante "Sì acconsento" potrà procedere alla compilazione del questionario.

Nelle pagine che seguono Le saranno descritte alcune prassi di ricerca.
Per ogni prassi Le sarà chiesto:

1. Di valutarne la prevalenza stimando la percentuale di psicologi ricercatori **che l'hanno adottata in almeno un'occasione.**
2. Di stimare la percentuale di chi, **tra questi psicologi ricercatori**, dichiarerebbe di averla adottata in almeno un'occasione.
3. Di dichiarare se **Lei abbia mai adottato questa prassi**.

Questi tre tipi di informazione ci aiuteranno a sviluppare una più accurata valutazione della diffusione di ciascuna prassi.

PRASSI: **In un articolo di ricerca, riportare in maniera selettiva solo gli studi che hanno "funzionato".**

Le chiediamo di stimare…

	0	10	20	30	40	50	60	70	80	90	100
La percentuale di psicologi ricercatori italiani che **hanno adottato** questa prassi:											
**Tra questi psicologi ricercatori**, la percentuale di coloro che dichiarerebbero di aver adottato questa prassi:											

Lei ha mai adottato questa prassi?

○ Sì

○ No

Ritiene che sia una prassi giustificabile?

○ No

○ In alcuni casi

○ Sì

PRASSI: In un articolo di ricerca, non riportare tutte le condizioni sperimentali di uno studio.

Le chiediamo di stimare…

	0	10	20	30	40	50	60	70	80	90	100
La percentuale di psicologi ricercatori italiani che **hanno adottato** questa prassi:											
**Tra questi psicologi ricercatori**, la percentuale di coloro che dichiarerebbero di aver adottato questa prassi:											

Lei ha mai adottato questa prassi?

○ Sì
○ No

Ritiene che sia una prassi giustificabile?

○ No
○ In alcuni casi
○ Sì

PRASSI: In un articolo di ricerca, non riportare tutte le misure dipendenti di uno studio.

Le chiediamo di stimare…

	0	10	20	30	40	50	60	70	80	90	100
La percentuale di psicologi ricercatori italiani che **hanno adottato** questa prassi:											
**Tra questi psicologi ricercatori**, la percentuale di coloro che dichiarerebbero di aver adottato questa prassi:											

Lei ha mai adottato questa prassi?

○ Sì

○ No

Ritiene che sia una prassi giustificabile?

○ No

○ In alcuni casi

○ Sì

PRASSI: Decidere di raccogliere ulteriori dati dopo aver controllato se i risultati sono significativi.

Le chiediamo di stimare…

	0	10	20	30	40	50	60	70	80	90	100
La percentuale di psicologi ricercatori italiani che **hanno adottato** questa prassi:											
**Tra questi psicologi ricercatori**, la percentuale di coloro che dichiarerebbero di aver adottato questa prassi:											

Lei ha mai adottato questa prassi?

○ Sì

○ No

Ritiene che sia una prassi giustificabile?

○ No

○ In alcuni casi

○ Sì

PRASSI: **Fermarsi nella raccolta dei dati prima del previsto perché si è già trovato il risultato atteso.**

Le chiediamo di stimare…

	0	10	20	30	40	50	60	70	80	90	100
La percentuale di psicologi ricercatori italiani che **hanno adottato** questa prassi:											
**Tra questi psicologi ricercatori**, la percentuale di coloro che dichiarerebbero di aver adottato questa prassi:											

Lei ha mai adottato questa prassi?

○ Sì

○ No

Ritiene che sia una prassi giustificabile?

○ No

○ In alcuni casi

○ Sì

PRASSI: **In un articolo di ricerca, riportare un risultato inatteso come se fosse stato previsto dall'inizio.**

Le chiediamo di stimare…

	0	10	20	30	40	50	60	70	80	90	100
La percentuale di psicologi ricercatori italiani che **hanno adottato** questa prassi:											
**Tra questi psicologi ricercatori**, la percentuale di coloro che dichiarerebbero di aver adottato questa prassi:											

Lei ha mai adottato questa prassi?

○ Sì
○ No

Ritiene che sia una prassi giustificabile?

○ No
○ In alcuni casi
○ Sì

PRASSI: **Falsare i dati.**

Le chiediamo di stimare…

	0	10	20	30	40	50	60	70	80	90	100
La percentuale di psicologi ricercatori italiani che **hanno adottato** questa prassi:											
**Tra questi psicologi ricercatori**, la percentuale di coloro che dichiarerebbero di aver adottato questa prassi:											

Lei ha mai adottato questa prassi?

○ Sì

○ No

Ritiene che sia una prassi giustificabile?

○ No

○ In alcuni casi

○ Sì

PRASSI: **Decidere se escludere o meno alcuni dati dopo aver visto l'impatto che ciò determina sui risultati.**

Le chiediamo di stimare…

	0	10	20	30	40	50	60	70	80	90	100
La percentuale di psicologi ricercatori italiani che **hanno adottato** questa prassi:											
**Tra questi psicologi ricercatori**, la percentuale di coloro che dichiarerebbero di aver adottato questa prassi:											

Lei ha mai adottato questa prassi?

○ Sì

○ No

Ritiene che sia una prassi giustificabile?

○ No

○ In alcuni casi

○ Sì

PRASSI: In un articolo di ricerca, "arrotondare" un p-value (ad esempio: riportare un p-value osservato = 0,054 come se fosse < 0,05).

Le chiediamo di stimare…

	0	10	20	30	40	50	60	70	80	90	100
La percentuale di psicologi ricercatori italiani che **hanno adottato** questa prassi:											
**Tra questi psicologi ricercatori**, la percentuale di coloro che dichiarerebbero di aver adottato questa prassi:											

Lei ha mai adottato questa prassi?

○ Sì

○ No

Ritiene che sia una prassi giustificabile?

○ No

○ In alcuni casi

○ Sì

PRASSI: **In un articolo di ricerca, affermare che le variabili demografiche (ad esempio: il genere) non influenzano i risultati quando in realtà si è incerti (o si è a conoscenza della loro influenza).**

Le chiediamo di stimare…

	0	10	20	30	40	50	60	70	80	90	100
La percentuale di psicologi ricercatori italiani che **hanno adottato** questa prassi:											
**Tra questi psicologi ricercatori**, la percentuale di coloro che dichiarerebbero di aver adottato questa prassi:											

Lei ha mai adottato questa prassi?

○ Sì

○ No

Ritiene che sia una prassi giustificabile?

○ No

○ In alcuni casi

○ Sì

Ritiene di avere dei dubbi sull'integrità della ricerca svolta da…

	Mai	Una o due volte	Occasionalmente	Spesso
Ricercatori di altre istituzioni				
Altri ricercatori della Sua istituzione				
Dottorandi / Assegnisti				
Suoi collaboratori				
Lei stesso/a				

A quale sezione AIP lei afferisce?
○ Psicologia clinica e dinamica
○ Psicologia per le organizzazioni
○ Psicologia dello sviluppo e dell'educazione
○ Psicologia sociale
○ Psicologia sperimentale

Qual è il suo Settore Scientifico Disciplinare:
○ M-PSI/01
○ M-PSI/02
○ M-PSI/03
○ M-PSI/04
○ M-PSI/05
○ M-PSI/06
○ M-PSI/07
○ M-PSI/08

Lei è:
○ Dottorando/a
○ Borsista/Assegnista di ricerca
○ Ricercatore universitario
○ Professore associato
○ Professore ordinario
○ Altro

Durante lo scorso anno ha mai avuto la tentazione di adottare almeno una delle 10 prassi elencate in precedenza al fine di aumentare la possibilità di pubblicazione e l'avanzamento della Sua carriera?
○ Mai
○ Una o due volte
○ Occasionalmente
○ Spesso

La ringraziamo per la Sua partecipazione.

Prima di terminare, Le chiediamo di riportare nel box sottostante commenti, domande o suggerimenti su questo questionario.
